# Influence of peer review on the reporting of primary outcome(s) and statistical analyses of randomised trials

**DOI:** 10.1186/s13063-017-2395-4

**Published:** 2018-01-11

**Authors:** Sally Hopewell, Claudia M. Witt, Klaus Linde, Katja Icke, Olubusola Adedire, Shona Kirtley, Douglas G. Altman

**Affiliations:** 1 Oxford Clinical Trials Research Unit, Nuffield Department of Orthopaedics, Rheumatology and Musculoskeletal Sciences, University of Oxford, Botnar Research Centre, Windmill Road, Oxford, OX3 7LD UK; 2 Centre for Statistics in Medicine, Nuffield Department of Orthopaedics, Rheumatology and Musculoskeletal Sceinces, University of Oxford, Botnar Research Centre, Windmill Road, Oxford, UK; 3 Institute for Complementary and Integrative Medicine, University of Zurich and University Hospital Zurich, Zurich, Switzerland; 4 Institute for Social Medicine, Epidemiology and Health Economics, Charité – Universitätsmedizin Berlin, Berlin, Germany; 5 Institute of General Practice, Technical University of Munich (TUM), TUM School of Medicine, Munich, Germany

**Keywords:** Randomised controlled trials, Selective outcome reporting, Peer review

## Abstract

**Background:**

Selective reporting of outcomes in clinical trials is a serious problem. We aimed to investigate the influence of the peer review process within biomedical journals on reporting of primary outcome(s) and statistical analyses within reports of randomised trials.

**Methods:**

Each month, PubMed (May 2014 to April 2015) was searched to identify primary reports of randomised trials published in six high-impact general and 12 high-impact specialty journals. The corresponding author of each trial was invited to complete an online survey asking authors about changes made to their manuscript as part of the peer review process. Our main outcomes were to assess: (1) the nature and extent of changes as part of the peer review process, in relation to reporting of the primary outcome(s) and/or primary statistical analysis; (2) how often authors followed these requests; and (3) whether this was related to specific journal or trial characteristics.

**Results:**

Of 893 corresponding authors who were invited to take part in the online survey 258 (29%) responded. The majority of trials were multicentre (*n* = 191; 74%); median sample size 325 (IQR 138 to 1010). The primary outcome was clearly defined in 92% (*n* = 238), of which the direction of treatment effect was statistically significant in 49%. The majority responded (1–10 Likert scale) they were satisfied with the overall handling (mean 8.6, SD 1.5) and quality of peer review (mean 8.5, SD 1.5) of their manuscript. Only 3% (*n* = 8) said that the editor or peer reviewers had asked them to change or clarify the trial’s primary outcome. However, 27% (*n* = 69) reported they were asked to change or clarify the statistical analysis of the primary outcome; most had fulfilled the request, the main motivation being to improve the statistical methods (*n* = 38; 55%) or avoid rejection (*n* = 30; 44%). Overall, there was little association between authors being asked to make this change and the type of journal, intervention, significance of the primary outcome, or funding source. Thirty-six percent (*n* = 94) of authors had been asked to include additional analyses that had not been included in the original manuscript; in 77% (*n* = 72) these were not pre-specified in the protocol. Twenty-three percent (*n* = 60) had been asked to modify their overall conclusion, usually (*n* = 53; 88%) to provide a more cautious conclusion.

**Conclusion:**

Overall, most changes, as a result of the peer review process, resulted in improvements to the published manuscript; there was little evidence of a negative impact in terms of post hoc changes of the primary outcome. However, some suggested changes might be considered inappropriate, such as unplanned additional analyses, and should be discouraged.

**Electronic supplementary material:**

The online version of this article (doi:10.1186/s13063-017-2395-4) contains supplementary material, which is available to authorized users.

## Background

Peer review is considered fundamental to the integration of new research findings into the scientific community [1]. Journal editors rely on the views of independent experts (peer reviewers) in making decisions on the publication of submitted manuscripts [2] and peer review is widely considered necessary for evaluating the credibility of published research. Worldwide, peer review costs an estimated £1.9 billion annually and accounts for around one quarter of the overall costs of scholarly publishing and distribution [3].

Despite this huge investment and widespread acceptance of the peer review process by the scientific community, little is known about its impact on the quality of reporting of published research [4, 5]. Studies have shown that peer reviewers are not able to appropriately detect errors [6–8], improve the completeness of reporting [9], or decrease the distortion of the study results [10]. One such study evaluating the impact of peer review found that although peer reviewers often detect important deficiencies in the reporting of the methods and results of randomised trials they miss more omissions than they detect [9]. That study showed that on average peer reviewers requested few changes. Most changes were deemed by the authors to have had a positive impact on reporting of the final publication; for example, clarification of the primary and secondary outcomes or the toning down of conclusions to reflect the results. However, some changes requested by peer reviewers were deemed inappropriate and could have a negative impact on reporting of the final publication, such as the adding of unplanned post hoc additional analyses.

In this study, we investigated the influence of the peer review process within biomedical journals on the reporting of primary outcome(s) and statistical analyses within published reports of randomised trials. In particular, we examined how often relevant post hoc changes of the primary outcome(s) and/or the primary statistical analysis were requested, how often the authors followed these requests, and whether the frequency of these suggestions was related to specific journal or trial characteristics.

## Methods

### Sample selection

We searched the US National Library of Medicine’s PubMed database over a 1 year period to identify all primary reports of randomised trials published between May 2014 to April 2015 in the six general medical journals and 12 specialty journals with the highest ISI impact factor in 2012. The six general medical journals were the *New England Journal of Medicine*, *Lancet*, *JAMA*, *BMJ*, *PLoS Medicine* and *Annals of Internal Medicine*. The top 12 specialty journals (Table 1) were identified from the major ISI Web of Knowledge journal citation reports medical subject categories. We restricted inclusion to journals that had published at least 50 articles in 2013 with the Publication Type term ‘Randomized Controlled Trial’ (based on a PubMed search on 7 April 2014) (see Additional file 1).

**Table 1:** Identification of primary reports of randomised controlled trials (RCTs) (May 2014 to April 2015) and number of authors responding to the online survey

Journal	Number of RCTs	Survey completed	% survey completed
General			
*Annals of Internal Medicine*	18	6	33%
*BMJ*	31	13	42%
*JAMA*	76	20	26%
*Lancet*	85	28	33%
*New England Journal of Medicine*	134	45	34%
*PLoS Medicine*	16	9	56%
Specialty			
*Anesthesia and Analgesia*	34	9	26%
*Annals of Rheumatic Diseases*	42	12	29%
*British Journal of Anaesthesia*	39	19	49%
*Circulation*	22	9	41%
*Diabetes Care*	84	12	14%
*Journal of the American College of Cardiology*	27	3	11%
*Journal of Clinical Oncology*	70	19	27%
*Journal of Infectious Diseases*	23	6	26%
*Journal of Pediatrics*	31	7	23%
*Lancet Oncology*	75	20	27%
*Pediatrics*	59	14	24%
*Stroke*	27	7	26%
Total	893	258	29%

We developed a search strategy based on a modified version of the Cochrane highly sensitive search strategy (see Additional file 2). The search was run monthly to identify reports of randomised trials published in the previous month (e.g. the search was performed in the third week of June 2014 for all articles published in the print version of each journal in May 2014). One reviewer (SH) screened the titles and abstracts of all retrieved reports to exclude any obvious reports of ineligible trials. A copy of the full article was then obtained for all non-excluded reports, and assessed by two reviewers to determine if it met the inclusion criteria.

### Eligibility criteria

We included all primary reports (that is, those reporting the main study outcome) of randomised trials, defined as a prospective study assessing healthcare interventions in human participants who were randomly allocated to study groups. We included all studies of parallel-group, crossover, cluster, factorial and split-body design. We excluded protocols of randomised trials, secondary analyses, systematic reviews, methodological studies, pilot studies and early phase (phase 1) trials.

### Online survey

We sent an invitation email (see Additional file 3) to the corresponding author of each eligible report of a randomised trial identified from our search. These authors were asked to participate in an online survey investigating the type of changes made to manuscripts of randomised trials as part of the peer review process. If they agreed to participate, an Internet link included in the invitation email gave them access to the online survey. Participants were asked to confirm that they agreed to participate before being able to complete the online survey. The online survey was tested by members of the study team to ensure comprehension of the survey questions.

The survey consisted of short questions, which asked them to rate on a 1–10 Likert scale the overall handling of their manuscript by the journal, the quality of the peer review process, and whether the editor or peer reviewers had asked them to change any aspects of their study in a way that deviated from what was planned in their trial protocol. They were also asked specific questions about changes to the primary outcome measure(s) and/or the analysis of the primary outcome, additional analyses not included in the original manuscript and whether they had been asked to modify or change their overall conclusions. Authors were asked to comment on the nature of the changes requested in free-text boxes (see Additional file 4).

The invitation emails were sent out in a batch each month, approximately 6 weeks after publication of the journal articles. We inserted the reference to their trial publication in the invitation email to ensure that we could link author responses to individual journal articles. Participants were informed that all responses would be treated in the strictest confidence and that we would not identify any individual responses. A reminder email was sent 2 weeks after the original email was sent, to those who did not respond to the original request. A second reminder was sent 2 weeks after sending the first reminder.

### Data extraction and analysis

Key information was extracted from each eligible report of a randomised trial for which the corresponding author had completed the online survey. After piloting of the data extraction form data extraction was carried out by one reviewer (OA); any uncertainties were resolved by discussion with a second reviewer (SH). We extracted information on the trial design, single or multicentre, number of study arms, number of participants, disease area, type of intervention, specification of the primary outcome and direction of the observed effect, trial registration and source of funding. We summarised the characteristics of the primary reports of randomised trials using proportions, median and interquartile range (IQR). Similarly, authors’ responses to the online survey were summarised using proportions, number, mean, standard deviation, median and interquartile range.

The primary analysis focussed on the responses to the online survey and the nature and extent of changes made to manuscripts by authors as part of the peer review process; in particular, in relation to the reporting of the primary outcome(s) and statistical analyses, how often the authors followed peer reviewer requests, and their main motivation for doing so. We assessed whether requested changes or modifications to the primary outcome(s) and statistical analysis were related to the type of journal (general/specialty), type of intervention (drug/non-drug), source of funding (industry/non-industry), or result of the primary outcome (significant/non-significant) using the chi-squared test or Fisher’s exact test as appropriate.

We also carried out a semi-qualitative analysis of free-text information provided by authors on changes and clarifications requested by peer reviewers and the extent to which authors responded to these requests. Two reviewers (CW, KL) first developed preliminary codes based on the questions posed and a screening of the free-text material. The free-text material was then systematically assigned to codes; if necessary codes were revised or new codes added.

## Results

The search identified 2106 possible reports of randomised trials published in the top six general medical journals and top 12 specialty journals between May 2014 and April 2015. After screening the full-text articles we identified 893 primary reports of randomised trials, for which 258 (29%) authors completed the online survey (Table 1). The response rate by journal ranged from 11% (*Journal of the American College of Cardiology*) to 56% (*PLoS Medicine*).

### Characteristics of primary reports of randomised trials

Table 2 shows the general characteristics of the 258 reports of randomised trials whose authors completed the online survey. The majority of trials were multicentre (*n* = 191; 74%), parallel group (*n* = 225; 87%), with two study groups (*n* = 202; 78%). The median sample size was 325 participants (IQR 138 to 1010). About half of the trials assessed drug interventions (*n* = 127; 49%), 23% (*n* = 59) surgical or procedural interventions and 16% (*n* = 40) behavioural or educational interventions. The primary outcome was clearly defined in 92% (*n* = 238) of trial reports, of which the estimated treatment effect was reported as statistically significant (*P* < 0.05) in 49% (*n* = 116). Most trials reported the trial registration number (*n* = 235; 91%) and around half (*n* = 141; 55%) gave a journal reference for the trial protocol; more than half (*n* = 159; 62%) were non-industry funded.

**Table 2:** Characteristics of primary reports of randomised trials (*n* = 258)

Characteristic	*n* = 258
Journal type	
General medical	118 (46%)
Specialty	140 (54%)
Most common specialties	
Oncology	44 (17%)
Cardiology	42 (16%)
Paediatrics	26 (10%)
Anaesthesia	22 (8%)
Public health	18 (7%)
Most common countries where trial was conducted	
USA	40 (22%)
UK	27 (15%)
Netherlands	16 (9%)
Australia	4 (2%)
Not reported	38 (20%)
Trial design^a^	
Parallel	225 (86.5%)
Cluster	16 (6%)
Factorial	11 (4%)
Crossover	8 (3%)
Split-body	1 (0.5%)
Intervention	
Drug	127 (49%)
Surgery/procedure	59 (23%)
Behavioural/education	40 (16%)
Biological/vaccine	12 (5%)
Device	9 (3%)
Dietary supplement	6 (2%)
Other	5 (2%)
Study centres	
Single	46 (18%)
Multiple	191 (74%)
Unclear	21 (8%)
Number of intervention groups	
2	202 (78%)
3	35 (13.5%)
4	20 (8%)
> 4	1 (0.5)
Median sample size (IQR)^b^	325 (138 to 1010)
10 to 90 percentile	(60 to 2485)
Primary outcome clearly defined	
Defined	238 (92%)
Not defined	20 (8%)
Statistical significance of primary outcome (where defined)^c^	
Significant	116 (49%)
Non-significant	122 (51%)
Trial registration	
Reported	235 (91%)
Not reported	23 (9%)
Trial protocol accessible	
Reported	141 (55%)
Not reported	117 (45%)
Source of funding	
Solely industry	52 (20%)
Part industry	27 (10%)
Non-industry	159 (62%)
No funding	7 (3%)
Not reported	13 (5%)

^a^More than one trial design is applicable (*n* = 3), ^b^Excluding cluster randomised trials (*n* = 242), ^c^Primary outcome clearly defined (*n* = 238). *IQR* interquartile range

### Response to online survey by authors of primary reports of randomised trials

Tables 3 and 4 summarise the authors’ responses to the online survey, and the nature and extent of changes made to manuscripts by authors as part of the peer review process in relation to the reporting of the primary outcome(s) and statistical analyses. The majority of authors responded that they were satisfied with the handling of their manuscript by the journal (mean 8.6, SD 1.5) and the quality of peer review (mean 8.5, SD 1.5). Fourteen percent (*n* = 36) of authors responded that the editor or peer reviewers asked them to change an aspect of their study in way that deviated from what was planned in the trial protocol.

**Table 3:** Authors’ responses to online survey (*n* = 258)

	Survey responses *n* = 258
1. How satisfied have you been with the overall handling of your manuscript by the journal? (1 = very unsatisfactory to 10 = very satisfactory)	
Mean (SD)	8.6 (1.5)
Median	9.0
Min – max	1–10
2. How would you rate the overall quality of the peer review of your manuscript? (1 = very low to 10 = very high)	
Mean (SD)	8.5 (1.5)
Median	9.0
Min – max	1–10
3. In getting your manuscript published, did the editor or peer reviewers ask you to change any aspects of your study so that it was different from what was planned in your trial protocol?	
Yes	36 (14%)
No	222 (86%)
4. Did the editor or peer reviewers ask you to change or clarify the trial’s primary outcomes measure(s)?	
Yes	8 (3%)
No	250 (97%)
5. Did the editors or peer reviewers ask you to change or clarify the statistical analysis of your primary outcome measure(s)?	
Yes	69 (27%)
No	189 (73%)
6. Were you asked to include any additional analyses that had not been included in the original manuscript?	
Yes	94 (36%)
No	164 (64%)
7. Were you asked to modify your overall conclusions?	
Yes	60 (23%)
No	198 (77%)
8. Did you register your trial in a trial registry?	
Yes	249 (96.5%)
No	9 (3.5%)
9. Did you publish the protocol of this trial in a journal?	
Yes	136 (53%)
No	122 (47%)

**Table 4:** Type of change or clarification requested to primary outcome and/or statistical analysis

Sub-questions	4a. Change or clarify the trial’s primary outcome measures? (yes, *n* = 8)	5a. Change or clarify the statistical analysis? (yes, *n* = 69)	6a. Include any additional analyses? (yes, n = 94)	7a. Modify the overall conclusion? (yes, *n* = 60)
Who requested the change? *n* (%)^a^				
Editor	3 (38%)	22 (32%)	29 (31%)	35 (58%)
Reviewer	8 (100%)	39 (57%)	77 (82%)	31 (52%)
Statistician	0 (0%)	32 (46%)	21 (22%)	6 (10%)
Do not know	0 (0%)	2 (3%)	0 (0%)	2 (3%)
What changes or clarifications were requested? *n* (%)^a^				
Change of the primary outcome measure	2 (25%)	n/a	n/a	n/a
Clarification of the primary outcome measure	2 (25%)	n/a	n/a	n/a
More cautious conclusion	n/a	n/a	n/a	53 (88%)
Stronger conclusion	n/a	n/a	n/a	2 (3%)
Other	4 (50%)	n/a	n/a	5 (8%)
Did you fulfil the request? *n* (%)				
Yes	5 (62.5%)	66 (96%)	83 (88%)	54 (90%)
No	3 (37.5%)	3 (4%)	11 (12%)	6 (10%)
What was your main motivation to fulfil the request? *n* (%)^a^				
Improvement of the reporting of the trial	4 (50%)	n/a	n/a	32 (53%)
Improvement of the statistical methods/analysis	n/a	38 (55%)	30 (32%)	n/a
Avoiding rejection of the paper	2 (25%)	30 (44%)	49 (52%)	29 (48%)
Other	3 (38%)	16 (23%)	31 (33%)	4 (7%)
How did you judge the request? (1 = not problematic to 10 = very problematic)				
Mean (SD)	6.8 (3.1)	3.6 (2.4)	3.7 (2.6)	3.5 (2.2)
Median	7.5	3.0	3.0	3.0
Range	2–10	1–10	1–10	1–10
Does the published article indicate that the analyses have not been pre-specified in the protocol? *n* (%)				
Yes	n/a	n/a	22 (23%)	n/a
No			72 (77%)	

^a^Multiple answers possible

Only eight authors (3%) said that the editor or peer reviewers asked them to change or clarify the trial’s primary outcome, but about a quarter (*n* = 69; 27%) reported that they had been asked to change or clarify the statistical analysis of the primary outcome. Most of those who were asked to change or clarify the trial’s primary outcome or statistical analysis responded that they had fulfilled the request. The main motivation for making the change to the statistical analysis was either to improve the statistical methods (*n* = 38; 55%) or to avoid rejection of paper (*n* = 30; 44%). Overall, there was no evidence of association between authors being asked to change or clarify the trial’s primary outcome and/or statistical analysis and whether the trial was published in a general or specialty journal, investigated a drug or was a non-drug intervention, or was solely/partly industry funded versus non-industry funded (Table 5). The primary outcome was more likely to be statistically significant where authors responded that they had been asked to change or clarify the trial’s primary outcome; however, the number of responses was very small (six out of eight responses; 86%). Conversely, requests for changes or clarification of the statistical analysis of the primary outcome were more common (40 out of 69 responses; 60%) for trials with non-significant primary outcomes.

**Table 5:** Association between whether editors or peer reviewers asked for changes or clarifications to the primary outcome(s) and/or statistical analysis and specific journal and trial characteristics

4. Did the editor or peer reviewers ask you to change or clarify the trial`s primary outcomes measure(s)?	Yes	No	Fisher’s exact test	*P* value
	(*n* = 8)	(*n* = 250)		
Type of journal				
General	3 (38%)	115 (46%)	0.73	0.46
Specialty	5 (62%)	135 (54%)		
Type of intervention				
Drug	2 (25%)	125 (50%)	0.28	0.15
Non-drug	6 (75%)	125 (50%)		
Statistical significance of primary outcome (where defined)^a^				
Significant	6 (86%)	110 (48%)	0.06	0.05
Non-significant	1 (14%)	121 (52%)		
Funding source^b^				
Sole/part industry	1 (14%)	78 (34%)	0.43	0.26
Non-industry	6 (86%)	153 (66%)		
5. Did the editors or peer reviewers ask you to change or clarify the statistical analysis of your primary outcome measure(s)?	Yes (*n* = 69)	No (*n* = 189)	Chi^2^ test	*P* value
Type of journal				
General	31 (45%)	87 (46%)	0.02	0.88
Specialty	38 (55%)	102 (54%)		
Type of intervention				
Drug	28 (41%)	99 (52%)	2.82	0.09
Non-drug	41 (59%)	90 (48%)		
Statistical significance of primary outcome (where defined)^a^				
Significant	27 (40%)	89 (52%)	2.66	0.10
Non-significant	40 (60%)	82 (48%)		
Funding source^b^				
Sole/part industry	17 (26%)	62 (36%)	1.20	0.16
Non-industry	48 (74%)	111 (64%)		

^a^Primary outcome clearly defined (*n* = 238)

^b^Sole/part industry funded and non-industry funded (*n* = 238)

One third (*n* = 94; 36%) of authors responded that they were asked to include additional analyses. Again, most authors (*n* = 83; 88%) had complied with the request with the main motivation being to avoid rejection of paper (*n* = 49; 52%). Most of the published articles (*n* = 72; 77%) did not indicate that the additional analyses had not been specified in the protocol. Finally, around a quarter (*n* = 60; 23%) of authors were asked to modify their overall conclusion, in most cases (*n* = 53; 88%) to provide a more cautious conclusion. Again, the majority fulfilled the request, the main motivations being to improve reporting of the trial (*n* = 32; 53%) or to avoid rejection of paper (*n* = 29; 48%).

### Textual analysis of author comments to the online survey questions

Examination of the free-text information provided by the eight authors who answered they were asked ‘to change or clarify the trials primary outcome measure(s)’ showed that only two referred to true ‘changes’. One author reported that editors and a peer reviewer “*asked us to present just one primary outcome, despite our protocol specifying two*” (trial 434); another that a reviewer asked that “*we change the primary outcome from average depression severity during time in trial to depression severity at the end of treatment*” (trial 451). In both cases authors successfully argued against these requests. Two further authors reported issues which are actually changes to the *statistical analysis* of the primary outcome (methods for imputation of missing data and adjustment; trials 478 and 584), two were requested to provide non-pre-specified sensitivity or additional analyses (trials 599 and 328). In the two remaining cases the short information provided suggested that the submitted manuscript had insufficiently defined the primary outcome (trials 396 and 513). True changes requested in the statistical analysis of the primary outcome most often concerned addition to or changes of methods to impute missing data (*n* = 14), or of the statistical model or test (*n* = 11), and less frequently to analysis populations or adjustment issues. More often, however, editors and reviewers requested clarification of statistical methods or presentations (see Table 6 for examples of free-text responses).

**Table 6:** Example of free-text answers provided by authors regarding changes or clarifications of the statistical analysis of the primary outcome

Changes of methods to impute missing data
“….they asked us to include the multiple imputation data for the primary outcome, which was a sensitivity analysis, in the abstract and up front in the results. We ensured that all of the data were presented and it made almost no difference to the results so we were OK with it.” (case 478) “Requested that we change report of primary outcome to use multiple imputation of missing data.” (case 454)
Change of statistical model/methods
“We were asked to use a different type of regression model and different independent variables than we had stated in our trial protocol.” (case 556)
“They recommended a different statistical approach. In the end we felt it was a better approach, and it did not change the overall intent or purpose of the study, but it was different than what we originally had planned in our protocol.” (case 450)
Analysis populations and adjustment issues
“To include all patients after randomisation in ITT and to delete the per-protocol analysis from the main text. Also, to present the baseline adjusted analysis as primary analysis and the analysis adjusted for unbalanced baseline characteristic as additional analysis.” (case 509)
Clarifications or presentation issues
“The editors requested clarification on a planned non-inferiority analysis.” (case 651)
“Clarify statistical methods used.” (case 563)
“Change mean and SD into median and 95% CI in case of skewed distributions.” (case 462)

*CI* confidence interval, *SD* standard deviation

Free-text responses to the question on additional analyses requested were typically short. The request most often mentioned explicitly (*n* = 29) was for additional subgroup analyses. A further 20 authors simply said they were asked for additional analyses. A smaller number of requests can be summarised as for sensitivity analyses to check the robustness of findings, or for presentation of additional data. Twenty authors had been asked to draw more cautious conclusions; a further 20 reported a variety of specific changes which did not make conclusions more positive or negative. Four others were asked to draw stronger negative conclusions, one to draw more positive conclusions in a trial not finding expected differences; the remainder did not give a reason.

## Discussion

### Summary of main findings

This study provides a unique opportunity to investigate the influence of the peer review process within high-impact medical and specialty journals on the reporting of primary outcome(s) and statistical analyses in reports of randomised trials. Textual analysis of author comments from the online survey provides insight into the types of changes or modifications made by authors as part of the peer review process and the rationale behind these changes.

In our study, most authors who took part in the online survey responded positively regarding the overall handling and overall quality of peer review of their manuscript. We found evidence of journal editors or peer reviewers asking authors to change or clarify the trial’s primary outcome, in only eight out of the 258 (3%) published reports where authors responded to the online survey, which is encouraging given concerns regarding bias associated with selective reporting of primary outcomes in favour of significant outcomes [11]. We found some evidence of authors being asked to make changes to the statistical analysis of the primary outcome; however, in most cases these requests were in fact for clarifications of the existing methods, the main motivation being to either improve reporting of the statistical methods or to avoid rejection by the journal. Overall, we found no association between authors being asked to make these changes and the type of journal, intervention, significance of the primary outcome, or funding source. Some authors reported being asked to modify their overall conclusion, in most cases to provide a more cautious conclusion avoiding spin in interpretation of the trial results [10]. However, some changes requested as part of the peer review process were more concerning, whereby authors were asked to include additional analyses, such as subgroup analyses, that had not been included in the original manuscript, of which the majority were not pre-specified in the trial protocol. This is concerning as these additional analyses could be driven by an existing knowledge of the data, or the interests of the reader, rather than the primary focus of the study [12]. While the Consolidated Standards of Reporting Trials (CONSORT) Statement does not preclude additional analyses being performed it does stress the importance of distinguishing those which were pre-specified in the trial protocol and those which are exploratory [13]. It is, therefore, concerning that most of the reported changes to analyses were not reported as post hoc.

### Comparison with other studies

We are not aware of other studies specifically looking at the impact of the peer review process on the reporting of the primary outcome and statistical analyses of randomised trials. Other studies on the impact of peer review have predominantly focussed on the editorial process, such as the use of reporting checklists, blinding of peer reviewers [14, 15], or the implementation of training strategies for peer reviewers [16]. A number of studies have looked at the selective reporting of primary outcomes [11] or switching of outcomes from either the trial registry [17–19], or trial protocol [20] and the published manuscript. The COMPare study [21] systematically tracked the switching of outcomes in 67 clinical trials published in the top five medical journals (October 2015 to January 2016) to compare trial outcomes reported in the trial registry or protocol with those reported in the published article. Of the 67 trials, they identified 354 pre-specified outcomes that were not reported in the published manuscript and 357 new non-pre-specified outcomes that were silently added. The COMPare study looked at all trial outcomes and did not differentiate between primary and secondary outcomes.

### Limitations

Our study has several limitations. First, despite sending two reminder emails our response rate to the online survey was still relatively low at 29% and, therefore, we do not know about those who did not respond. One might expect both that those with more negative experiences might have been more likely to respond to the survey; or that people who experienced a positive spin after peer review were less likely to respond. Yet, our survey showed that most authors were happy with overall quality and handling of their manuscript as part of the peer review progress. Conversely, it is possible that our findings might present an overly positive picture as we focussed on authors whose manuscript had recently been accepted by a high-impact journal. Open peer review, whereby the peer reviewer’s comments are included alongside the published article would allow a more complete assessment of this problem, without the challenge of low response rates.

Due to the nature of our study, we do not have information on the influence of peer review on the reporting of primary outcome(s) and statistical analyses for those manuscripts which were subsequently rejected by the journal. Finally, our study focussed specifically on high-impact journals where one might expect the journal editors and peer reviewers to be more experienced and more likely to identify potential problems. It is unclear the extent to which these findings are generalizable to other journals with potentially less experienced editors and peer reviewers.

## Conclusion

Overall, we found little evidence of a negative impact of the peer review process in terms of selective reporting of primary outcome(s). Most changes requested as part of the peer review process resulted in improvements to the trial manuscript, such as improving clarity of the statistical methods and providing more cautious conclusions. However, some requested changes could be deemed inappropriate and could have a negative impact on reporting in the final publication, such as the request to add non-pre-specified additional analyses.

## Additional files

Additional file 1: Journal impact factor. (DOCX 36 kb)

Additional file 2: Search strategy for the PubMed database available from the US National Library of Medicine, National Institutes of Health. (DOCX 33 kb)

Additional file 3: Invitation email to survey participants. (DOCX 67 kb)

Additional file 4: Survey questions. (DOCX 35 kb)

## Abbreviations

CONSORT: Consolidated Standards of Reporting Trials

## References

[1] Rennie R. Editorial peer review: its development and rationale. In: Godlee F, Jefferson T, editors. Peer Review in Health Sciences. 2nd edition. London: BMJ Books; 2003. p. 1-13.

[2] Jefferson T, Rudin M, Brodney Folse S, Davidoff F. Editorial peer review for improving the quality of reports of biomedical studies. Cochrane Database Syst Rev. 2007;(2):Mr000016. 10.1002/14651858.MR000016.pub3 PMC8973931 17443635

[3] Public Library of Science (2011). Peer review—optimizing practices for online scholarly communication. House of Commons Science and Technology Committee, editor. Peer Review in Scientific Publications, Eighth Report of Session 2010–2012.

[4] Bruce R, Chauvin A, Trinquart L, Ravaud P, Boutron I (2016). Impact of interventions to improve the quality of peer review of biomedical journals: a systematic review and meta-analysis. BMC Med.

[5] Chauvin A, Ravaud P, Baron G, Barnes C, Boutron I (2015). The most important tasks for peer reviewers evaluating a randomized controlled trial are not congruent with the tasks most often requested by journal editors. BMC Med..

[6] Baxt WG, Waeckerle JF, Berlin JA, Callaham ML (1998). Who reviews the reviewers? Feasibility of using a fictitious manuscript to evaluate peer reviewer performance. Ann Emerg Med.

[7] Kravitz RL, Franks P, Feldman MD, Gerrity M, Byrne C, Tierney WM (2010). Editorial peer reviewers’ recommendations at a general medical journal: are they reliable and do editors care?. PloS One.

[8] Yaffe MB (2009). Re-reviewing peer review. Sci Signaling.

[9] Hopewell S, Collins GS, Boutron I, Yu LM, Cook J, Shanyinde M (2014). Impact of peer review on reports of randomised trials published in open peer review journals: retrospective before and after study. BMJ (Clinical research ed).

[10] Boutron I, Dutton S, Ravaud P, Altman DG (2010). Reporting and interpretation of randomized controlled trials with statistically nonsignificant results for primary outcomes. JAMA.

[11] Chan AW, Hrobjartsson A, Haahr MT, Gotzsche PC, Altman DG (2004). Empirical evidence for selective reporting of outcomes in randomized trials: comparison of protocols to published articles. JAMA.

[12] Sun X, Briel M, Busse JW, You JJ, Akl EA, Mejza F (2012). Credibility of claims of subgroup effects in randomised controlled trials: systematic review. BMJ (Clinical research ed).

[13] Moher D, Hopewell S, Schulz KF, Montori V, Gotzsche PC, Devereaux PJ (2010). CONSORT 2010 explanation and elaboration: updated guidelines for reporting parallel group randomised trials. BMJ (Clinical research ed).

[14] Alam M, Kim NA, Havey J, Rademaker A, Ratner D, Tregre B (2011). Blinded vs. unblinded peer review of manuscripts submitted to a dermatology journal: a randomized multi-rater study. Br J Dermatol.

[15] Cho MK, Justice AC, Winker MA, Berlin JA, Waeckerle JF, Callaham ML (1998). Masking author identity in peer review: what factors influence masking success? PEER Investigators. JAMA.

[16] Schroter S, Black N, Evans S, Carpenter J, Godlee F, Smith R (2004). Effects of training on quality of peer review: randomised controlled trial. BMJ (Clinical research ed).

[17] Mathieu S, Boutron I, Moher D, Altman DG, Ravaud P (2009). Comparison of registered and published primary outcomes in randomized controlled trials. JAMA.

[18] Hannink G, Gooszen HG, Rovers MM (2013). Comparison of registered and published primary outcomes in randomized clinical trials of surgical interventions. Ann Surg.

[19] Rosenthal R, Dwan K (2013). Comparison of randomized controlled trial registry entries and content of reports in surgery journals. Ann Surg.

[20] Dwan K, Gamble C, Williamson PR, Kirkham JJ (2013). Systematic review of the empirical evidence of study publication bias and outcome reporting bias—an updated review. PloS One.

[21] Goldacre B, Drysdale H, Powell-Smith A, et al. The COMpare Trials Project. 2016. http://compare-trials.org/. Accessed 27 Nov 2017.

