# Supplementary material for: Influence of peer review on the reporting of primary outcome(s) and statistical analyses of randomised trials
Source: Trials. 2018 Jan 11;19:30. doi: 10.1186/s13063-017-2395-4 (PMC5765661; doi:10.1186/s13063-017-2395-4)
Supplement: Supplementary file 1 — Journal impact factor. (DOCX 36 kb) [file 13063_2017_2395_MOESM1_ESM.docx]

**Additional file 1: Journal impact factor**

General medicine journals:

This group includes the six journals with the highest 2012 ISI Impact Factors in the category ‘general internal medicine’.

| Journal | Impact factor in 2012 | Number RCTs in 2013 |
| --- | --- | --- |
| New England Journal of Medicine | 51.66 | 121 |
| Lancet | 39.01 | 86 |
| JAMA | 29.98 | 78 |
| BMJ | 17.22 | 45 |
| PLoS Medicine | 15.25 | 2 |
| Annals of Internal Medicine | 13.98 | 19 |

Specialty journals:

The top 12 specialty journals with the highest ISI impact factor in 2012 were identified from the major ISI Web of Knowledge journal citation reports medical subject categories (anaesthesiology, cardiac and cardiovascular systems, clinical neurology, critical care medicine, endocrinology and metabolism, gastroenterology and hepatology, infectious diseases, obstetrics and gynaecology, oncology, pediatrics, peripheral vascular disease, psychiatry, respiratory medicine, rheumatology, surgery). In addition, they had to have published at least 50 articles in 2013 with the Publication Type term “Randomized Controlled Trial” (based on a PubMed search on 7 April 2014).

| Journal | Impact factor in 2012 | Number RCTs in 2013 | Rank |
| --- | --- | --- | --- |
| Journal of Clinical Oncology | 18.04 | 163 | 5^th^ in oncology |
| Journal of the American College of Cardiology | 14.09 | 102 | 3^rd^ in cardiac and cardiovascular systems |
| Pediatrics | 5.12 | 99 | 2^nd^ in pediatrics |
| Lancet Oncology | 24.12 | 74 | 3^rd^ in oncology |
| Diabetes Care | 7.74 | 70 | 7^th^ in endocrinology and metabolism |
| Stroke | 6.16 | 68 | 7^th^ in peripheral vascular disease |
| Annals of Rheumatic Diseases | 9.11 | 68 | 2^nd^ in rheumatology |
| Circulation | 15.20 | 65 | 1^st^ in cardiac and cardiovascular systems |
| Journal of Pediatrics | 4.04 | 61 | 4^th^ in pediatrics |
| Anesthesia and Analgesia | 3.3 | 57 | 6^th^ in anesthesiology |
| British Journal of Anaesthesia | 4.24 | 55 | 3^rd^ in anesthesiology |
| Journal of Infectious Diseases | 5.85 | 51 | 5^th^ in infectious diseases |
