# Supplementary material for: Influence of peer review on the reporting of primary outcome(s) and statistical analyses of randomised trials
Source: Trials. 2018 Jan 11;19:30. doi: 10.1186/s13063-017-2395-4 (PMC5765661; doi:10.1186/s13063-017-2395-4)
Supplement: Supplementary file 2 — Search strategy for the PubMed database available from the US National Library of Medicine, National Institutes of Health. (DOCX 33 kb) [file 13063_2017_2395_MOESM2_ESM.docx]

**Additional file 2: Search strategy for the PubMed database available from the US National Library of Medicine, National Institutes of Health**

1. randomized controlled trial [pt] OR controlled clinical trial [pt] OR randomized [tiab] OR randomised [tiab] OR placebo [tiab] OR randomly [tiab] OR trial [tiab]

2. "N Engl J Med"[Journal] OR "Lancet"[Journal] OR "JAMA"[Journal] OR "BMJ"[Journal] OR "PLoS Med"[Journal] OR "Ann Intern Med"[Journal] OR "J Clin Oncol"[Journal] OR "J Am Coll Cardiol"[Journal] OR "pediatrics"[journal] OR "Lancet Oncol"[Journal] OR "Diabetes Care"[Journal] OR "Stroke"[Journal] OR "Ann Rheum Dis"[Journal] OR "Circulation"[Journal] OR "J Pediatr"[Journal] OR "Anesth Analg"[Journal] OR "Br J Anaesth"[Journal] OR "J Infect Dis"[Journal]

3. "2014/05/01"[PDAT] : "2014/05/31"[PDAT]

4. #1 AND #2 AND #3

5. systematic review [ti] OR meta-analysis [ti] OR meta analysis [ti] OR review [ti] OR Review [pt] OR Meta-Analysis [pt] OR Comment [pt] OR Letter [pt] OR Editorial [pt] OR News [pt]

6. #4 NOT #5

7. #6 NOT pubstatusaheadofprint [all]

*Line 3 of the search strategy was revised each month to reflect the search conducted every month between May 2014 and April 2015*
