# Supplementary material for: Influence of peer review on the reporting of primary outcome(s) and statistical analyses of randomised trials
Source: Trials. 2018 Jan 11;19:30. doi: 10.1186/s13063-017-2395-4 (PMC5765661; doi:10.1186/s13063-017-2395-4)
Supplement: Supplementary file 3 — Invitation email to survey participants. (DOCX 67 kb) [file 13063_2017_2395_MOESM3_ESM.docx]

**Additional file 3: Invitation email to survey participants**

Influence of medical journals on the reporting of primary outcome(s) and statistical analyses of randomised trials

Dear [insert corresponding author]

We are interested in your experiences of the peer review process, we all know that the process can be very different for different journals.

We would like to invite you to participate in an international academic study to investigate the type of changes made to manuscripts of randomised controlled trials as part of the peer review process.

The study has been designed so that it will require only a minimal amount of work on your part and yet provides a real opportunity to actively contribute to improve the communication of research results to inform clinical practice.

Your participation would involve answering a series of short questions about the peer review process and publication of your recent study [insert publication] and its findings. The survey should take no more than 10 - 15 minutes of your time.

You can complete the survey by clicking on the following link:

[www.soscisurvey.de/journalsurvey/](http://www.soscisurvey.de/journalsurvey/)

All responses will be treated in the strictest confidence and we will not identify any individual responses in any resulting publications.

Thank you for your participation.


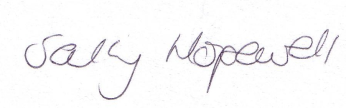


CAPRI 2 (Changes After Peer RevIew) study  investigators: Doug Altman (University of Oxford), Sally Hopewell (University of Oxford), Klaus Linde (Universität Munchen), Claudi Witt (Universitätsmedizin Berlin).

This study has been approved by the University of Oxford Central University Research Ethics Committee.
