# Supplementary material for: Influence of peer review on the reporting of primary outcome(s) and statistical analyses of randomised trials
Source: Trials. 2018 Jan 11;19:30. doi: 10.1186/s13063-017-2395-4 (PMC5765661; doi:10.1186/s13063-017-2395-4)
Supplement: Supplementary file 4 — Survey questions. (DOCX 35 kb) [file 13063_2017_2395_MOESM4_ESM.docx]

**Additional file 4: Survey questions**

1. How satisfied have you been with the overall handling of your manuscript by the journal?

2. How would you rate the overall quality of the peer review of your manuscript?

*Very low/very high (NRS 1-10)*

3. In getting your manuscript published, did the editors or peer reviewers ask you to change any aspects of your study so that it was different from what was planned in your trial protocol?

*Yes/No*

If yes, please provide details

*free text*

*In the next two questions we would like to ask you to provide information about any changes to the primary outcome measure(s) (question 4) and /or the primary analyses of the primary outcome measure (question 5).*

4a. Did the editors or peer reviewers ask you to change or clarify the trial’s primary outcome measure(s)?

*Yes/No*

If yes:

b. Who requested the change? *editor, reviewer, statistician, don’t know*

c. What changes or clarifications were requested?

*Change of the primary outcome measure (e.g. from days with headache to days with migraine)/ Clarification of the primary outcome measure (e.g. if you had listed multiple outcomes) / Other (free text)*

d. Did you fulfill the request? *Yes/No*

If yes, please provide details on the changes *free text*

e. What was your main motivation to fulfill the request?

*Improvement of the reporting of the trial/ Avoiding rejection of the paper /Other*

f. How did you judge the request: *not problematic at all / very problematic*

*(NRS 1-10)*

g. Please feel free to provide further comments *free text*

5a. Did the editors or peer reviewers ask you to change or clarify the statistical analysis of your primary outcome measure(s)?

*Yes/No*

If yes:

b. Who requested the change? *editor, reviewer, statistician, don’t know*

c. Please provide details of the requested changes *free text*

d. Did you fulfill the request? *Yes/No*

If yes, please provide details on the changes *free text*

e. What was your main motivation to fulfill the request?

*Improvement of the statistical methods / Avoiding rejection of the paper /Other*

f. How do you judge the request: *very problematic / not problematic at all*

g. Please feel free to provide further comments *free text*

6a. Were you asked to include any additional analyses that had not been included in the original manuscript? *Yes/No*

If yes:

b. Who requested the additional analyses? *editor, reviewer, statistician, don’t know*

c. Please provide details of the requested changes *free text*

d. Did you fulfill the request? *Yes/No*

If yes, please provide details on the changes *free text*

e. What was your main motivation to fulfill the request?

*Improvement of the statistical analysis***/** *avoiding rejection of the paper /other*

f. How do you judge the request: *not problematic at all / very problematic*

*(NRS 1-10)*

g. Does the published article indicate that the analyses have not been pre-specified in the protocol? *Yes/No*

h. Please feel free to provide further comments *free text*

7a. Were you asked to modify your overall conclusions? *Yes/No*

If yes:

b. Who requested the change? *editor, reviewer, don’t know*

c. What changes were requested?

*More cautious conclusion / Stronger conclusion / Other (free text)*

d. Did you fulfill the request? *Yes/No*

If yes, please provide details on the changes *free text*

e. What was your main motivation to fulfill the request?

*Improvement* **/** *Avoiding rejection of the paper /Other*

f. How do you judge the request: *not problematic at all / very problematic*

*(NRS 1-10)*

g. Please feel free to provide further comments *free text*

8. Did you register your trial in a trial registry (e.g. clinicaltrials.gov) *Yes/No*

9. Did you publish the protocol of this trial in a journal? *Yes/No*

10. Please feel free to add further aspects you would like to share

*free text*
